# Development of a New Robotic Ankle Rehabilitation Platform for Hemiplegic Patients after Stroke

**DOI:** 10.1155/2018/3867243

**Published:** 2018-03-15

**Authors:** Quanquan Liu, Chunbao Wang, Jian Jun Long, Tongyang Sun, Lihong Duan, Xin Zhang, Bo Zhang, Yajing Shen, Wanfeng Shang, Zhuohua Lin, Yulong Wang, Jinfeng Xia, Jianjun Wei, Weiguang Li, Zhengzhi Wu

**Affiliations:** ^1^Shenzhen Institute of Geriatrics, Shenzhen, China; ^2^The Second People's Hospital of Shenzhen, Shenzhen, China; ^3^The School of Mechanical & Automotive Engineering, South China University of Technology, Guangzhou, China; ^4^Faculty of Science and Engineering, Waseda University, Tokyo, Japan; ^5^The School of Mechanical Engineering, Guangxi University of Science and Technology, Liuzhou, China; ^6^The Beijing Advanced Innovation Center for Intelligent Robots and Systems, Beijing Institute of Technology, Beijing, China; ^7^Mechanical and Biomedical Engineering Department, City University of Hong Kong, Hong Kong

## Abstract

A large amount of hemiplegic survivors are suffering from motor impairment. Ankle rehabilitation exercises act an important role in recovering patients' walking ability after stroke. Currently, patients mainly perform ankle exercise to reobtain range of motion (ROM) and strength of the ankle joint under a therapist's assistance by manual operation. However, therapists suffer from high work intensity, and most of the existed rehabilitation devices focus on ankle functional training and ignore the importance of neurological rehabilitation in the early hemiplegic stage. In this paper, a new robotic ankle rehabilitation platform (RARP) is proposed to assist patients in executing ankle exercise. The robotic platform consists of two three-DOF symmetric layer-stacking mechanisms, which can execute ankle internal/external rotation, dorsiflexion/plantarflexion, and inversion/eversion exercise while the rotation center of the distal zone of the robotic platform always coincides with patients' ankle pivot center. Three exercise modes including constant-speed exercise, constant torque-impedance exercise, and awareness exercise are developed to execute ankle training corresponding to different rehabilitation stages. Experiments corresponding to these three ankle exercise modes are performed, the result demonstrated that the RARP is capable of executing ankle rehabilitation, and the novel awareness exercise mode motivates patients to proactively participate in ankle training.

## 1. Introduction

Recently, a large number of stroke survivors are suffering from motor impairment. The recovery of motor loss function is difficult conducted only by biomedical treatment [1]. Generally, ankle rehabilitation after stroke consists of three stages [2]. In the first stage, known as the first month after stroke, patients mainly play simple and passive training in bed. In the second stage, patients begin to participate in active rehabilitation exercises, including balance and coordination training to strengthen the affected ankle. In the third stage, patients try to reobtain their healthy state through intensive rehabilitation training. The first two stages aim to avoid patient muscle atrophy, and the third stage focuses on improving patients' life quality. To stroke survivors, rehabilitation at the first two stages plays an important role in recovering their walk ability by exercising the affected muscles [3, 4].

In the traditional ankle exercise, physiotherapy (PT) manually holds patients' affected ankle to carry out internal/external rotation, dorsiflexion/plantarflexion, and inversion/eversion motion during ankle rehabilitation. This manual training method makes PT exhausted, and the rehabilitation performance highly relies on a physiotherapist's experience. Furthermore, patients may act confrontation on this passive training method which reduces the rehabilitation efficiency. In order to address the manipulation challenges in ankle rehabilitation, many studies have considered the development of robotic systems to reduce a physiotherapist's workload and enhance patients' rehabilitation performance. A parallel structure robotic system and an exoskeletal structure robotic system are the two main research interests in the ankle rehabilitation assistance.

Saglia et al. reported a two-DOF foot pedal using parallel structure for ankle rehabilitation [2]. It can achieve ankle dorsiflexion/plantarflexion and inversion/eversion exercises under position control for patient-passive exercise mode or under admittance control for patient-active exercise mode. Rutgers University developed a 6-DOF ankle rehabilitation robot “Rutgers ankle” based on a Stewart platform driven by pneumatic actuation, where a 6-DOF force sensor was used to provide force and torque feedback generated between the patient's foot and the foot pedal [5]. Liu et al. announced a 3-RSS/S parallel robotic platform associated with a virtual training environment, which employed servo motor to drive its parallel links [6]. Meng et al. constructed a parallel structure ankle rehabilitation robot, where the patient's leg is fixed to the base of the robot; therefore, the robot can drive the patient's foot to generate relative movement using the ankle joint as the pivot center [7]. Muhammad and Shafriza presented a mechanical design and kinematic analysis of a parallel robot for ankle rehabilitation [8]. Yu et al. presented a 3-DOF cable-driven parallel robot for ankle rehabilitation. The mechanical design ensured that the mechanism center of the rotations can match the ankle axes of rotations [9].

Besides the parallel structure robotic ankle rehabilitation robot, a number of exoskeletal structure ankle rehabilitation robots were also developed. Jeffrey et al. presented a powered ankle-foot orthosis. The device owns only one DOF to realize ankle dorsiflexion/plantarflexion training [10]. Delaware University developed a wearable exoskeletal ankle rehabilitation robot to assist dorsiflexion/plantarflexion and inversion/eversion training [11]. Rahman and Ikeura developed a dynamic knee-ankle-foot orthosis to offer variable-impedance dorsiflexion/plantarflexion training using a wrap-spring clutch [12]. Hong et al. introduced a 3-DOF ankle rehabilitation robot, which consisted of a parallel connection of a spherical five-bar linkage and a revolute-spherical-universal serial chain to assist ankle dorsiflexion/plantarflexion and inversion/eversion training [13].

The summary of the existing state-of-the-art parallel structure ankle rehabilitation robots and exoskeletal ankle rehabilitation robots can be found in Table 1.

**Figure 1:**
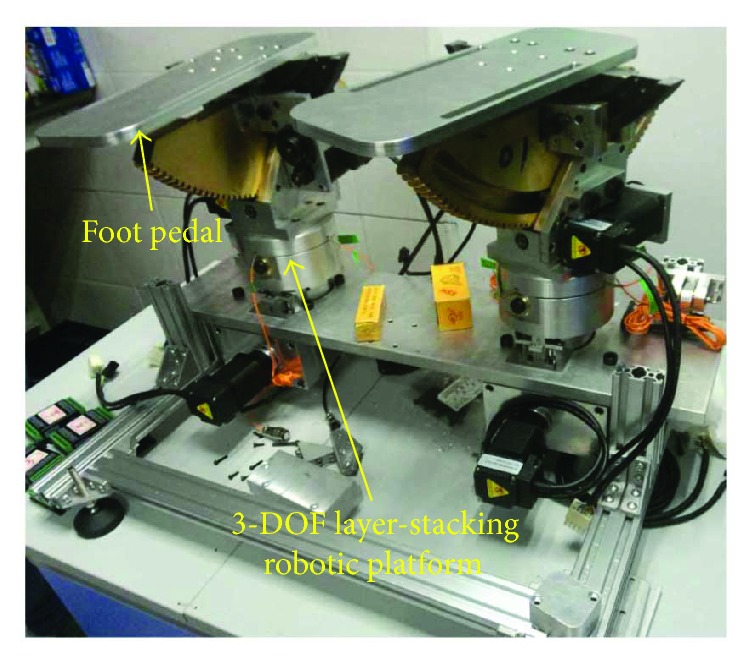
Constructed ankle rehabilitation robotic platform.

Through examining the existing robots for ankle rehabilitation, a few improvements could still be identified as follows: (1) the rotation center of the foot pedal should easily point to the rotation center of the ankle joint via mechanical configuration; (2) the robot should be friendly and stimulate the patient to participate in ankle training, encouraging the patient to self-balance both their ankle joints during exercise; and (3) maintainability and modularity might be improved. Trying to achieve these improved specifications, we developed a novel robotic ankle rehabilitation platform (RARP) for hemiplegic stroke survivors, as shown in Figure 1. It consists of two 3-DOF symmetric layer-stacking structure platforms; therefore, the mechanical configuration allows the patient to use his/her sound side ankle joint to teach the affected side ankle joint for ankle exercises under a physiotherapist's instructions.

This paper is organized as follows. The design specification of the RARP is presented in Section 2.1, while Section 2.2 is dedicated to the mechanical design of the RARP. And the control architecture of the RARP is described in Section 2.4. The experimental results are reported in Section 3, while the discussion is in Section 3.4. Finally, the conclusions are reported.

## 2. Materials and Methods

### 2.1. Design Specification of the RARP

**Figure 2:**
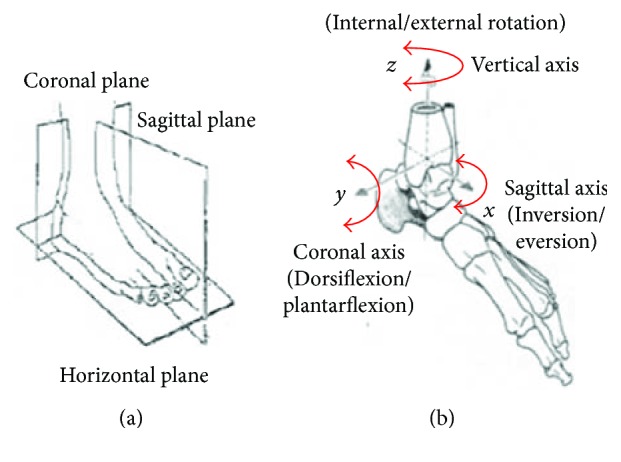
The anatomical planes and terms of location and orientation; (a) projection planes; (b) rotational axes.

Figure 2 demonstrates the anatomical characteristics of the human ankle joint. The rotation of the ankle joint can be projected to three orthogonal planes and divided into three different rotating movements with the corresponding axes. The ankle joint can be simplified as a spherical joint, and the rotation along the *x*-axis is to achieve inversion/eversion movement, while the rotation along the *y*-axis is to obtain dorsiflexion/plantarflexion movement and the rotation along the *z*-axis is to realize internal/external rotation. Considering the individual difference of ankle physiological characteristics, the range of each ankle motion is shown in Table 2 [14, 15].

### 2.2. Design Consideration and Iterations

The final design of the RARP mechanism was obtained after several rounds of design iterations. The following considerations led to the convergence of the final design.

#### 2.2.1. Exercise Mode

Corresponding to the different sources of tractive force for ankle movement, ankle rehabilitation can be sorted into patient-passive exercise and patient-active exercise. In the early stage of ankle therapy, the patient can hardly self-move his/her foot; therefore, a passive exercise with constant-speed movement to avoid muscle atrophy is necessary. This kind of task can be accomplished after the patient has carried out delicate movement by himself/herself. In order to allow the patient to fully reobtain his/her motor function, constant torque-impedance exercises can be executed with the device to enhance the strength of the patient's affected muscles.

To the above exercise modes, researchers pay most attentions to recover the range of motion and force strength of the patient's affected ankle; however, ankle exercise content should consider the balance and the coordination between the sound side ankle and the affected side ankle due to the individual differences. Hence, in this paper, we propose a new ankle exercise mode, named as awareness exercise that allows the patient to train his/her affected side ankle using the motor parameters obtained from his/her sound side ankle. With this exercise mode, the motor capability of the affected side ankle will approach that of the sound side ankle; therefore, it allows the patient to regain symmetric balance capability for walking after ankle exercise.

#### 2.2.2. Mechanical Configuration

**Figure 3:**
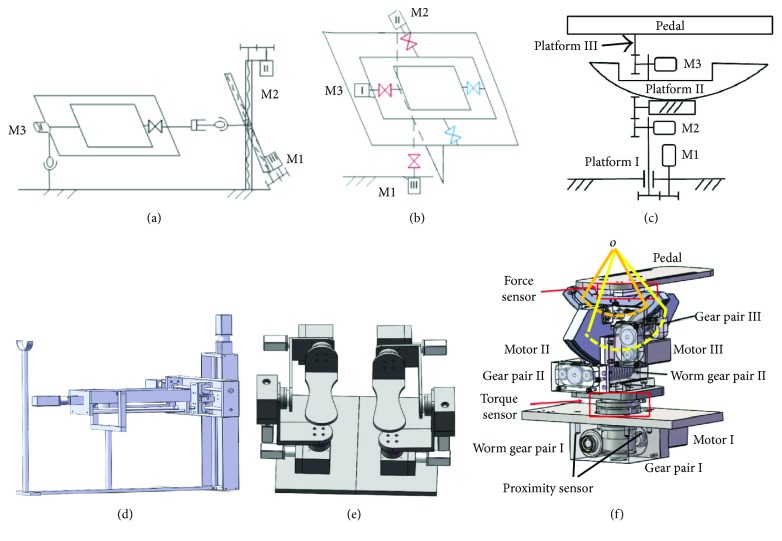
Three design solutions of the 3-DOF ankle rehabilitation robotic platform: (a) mechanical diagram of solution I; (b) mechanical diagram of solution II; (c) mechanical diagram of solution III; (d) 3D model of solution I; (e) 3D model of solution II; (f) 3D model of solution III.

A 3-DOF robotic platform is proposed to assist the ankle to achieve inversion/eversion, dorsiflexion/plantarflexion, and internal/external rotation. As shown in Figure 2(b), the joints of inversion/eversion and dorsiflexion/plantarflexion are independent, while located behind the movement of internal/external rotation. Three mechanical structures to realize 3-DOF ankle exercises are illustrated in Figure 3.

In the first design solution (Figure 3(a)), the rotation generated by motor M1 and motor M2 will be transferred to linear motion through a ball screw pair mechanism and then drives the foot pedal to obtain internal/external rotation and dorsiflexion/plantarflexion exercise, respectively. Motor M3 is directly mounted to the pedal mechanism; therefore, the rotation generated by M3 will drive the ankle joint to execute inversion/eversion ankle exercise.

In the second design solution (Figure 3(b)), the 3-DOF mechanism is constructed based on a serial mechanism structure. The coronal axis and the sagittal axis are located in the same plane to form a cross structure, while driven by motors M2 and M3, respectively. The base of motor M2 is fixed at the output shaft of motor M1; therefore, the rotation of motor M1 will drive the whole distal mechanism movement. Careful geometric dimensional design of the robotic platform can allow that the pivot point of the ankle joint coincides with the rotation center of the pedal.

In the third design solution (Figure 3(c)), the 3-DOF mechanism is stacked up layer by layer. In order to ensure that the rotation center of the pedal always points to the pivot center of the ankle joint, a circular plate is fitted in the worm gear pair mechanism; therefore, the rotation of the worm will drive the circular plate to rotate along its central axis. Since platform II and platform III are orthogonally mounted with each other, the rotation center of the foot pedal will automatically point to the pivot center of the ankle joint (Figure 3(f)). A torque sensor (FT20, Forsentek Co., Shenzhen, China) is mounted at the mechanism for internal/external rotation exercise, and two force sensors (FL25-100 kg, Forsentek Co., Shenzhen, China) are mounted under the foot pedal to detect the interaction force between patients' foot and the foot pedal.

All the three design solutions can accomplish ankle exercises with motion of inversion/eversion, dorsiflexion/plantarflexion, and internal/external rotation. In the first solution, the use of a screw pair mechanism can increase systematic rigidity; however, the volume is hard to be compacted. In the second solution, although the serial mechanism is easy for a compact design, the payload on the pedal will generate a large torque at the serial structure robotic joint; hence, high-torque motor is needed to execute ankle exercise and the cantilever needs to be strengthened. In the third solution, the rotation of the motor passes through the combination of gear pairs to drive the circular plate rotating along its central axis. This layer-stacking mechanism design takes use of the advantage that the high-torque transmission of worm gear pair and the payload on the pedal can be transmitted to the ground through the circular plates. Finally, the third solution is used in our robotic platform.

### 2.3. Component Description

As shown in Figure 3, the ankle rehabilitation robotic platform consists of three parts corresponding to inversion/eversion, dorsiflexion/plantarflexion, and internal/external rotation, respectively. We employ the combination of the gear pairs to execute a power transmission from the motor to the actuator.

#### 2.3.1. Mechanism for Internal/External Rotation Exercise

**Figure 4:**
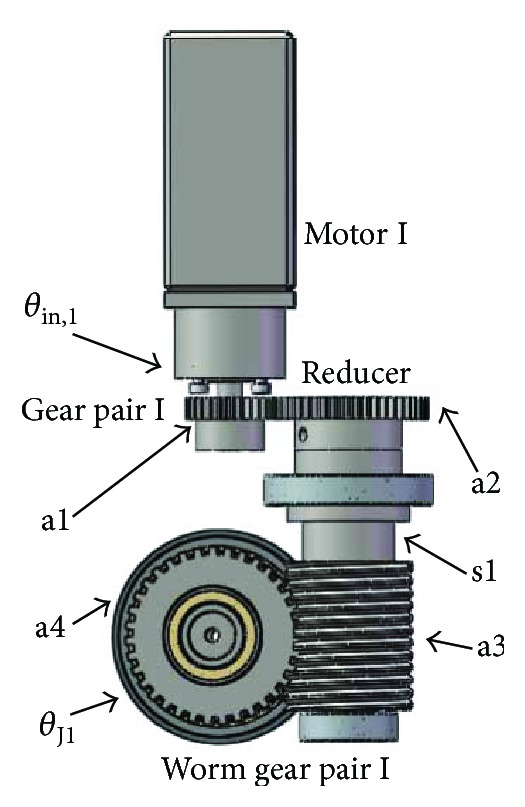
Components of the power transmission mechanism for internal/external rotation exercise.

The mechanism that activates ankle exercise of internal/external rotation (Figure 4) consists of gear pair I and worm gear pair II. Spur gear a1 transmits motor's rotation to the shaft s1 through gear pair I. Due to spur a2 and worm a3 being coaxial, the mechanism allows us to transmit the mechanical power to the worm gear pair I. Define the gear ratio *N*_*i*,*j*_ between two consecutive gears as

(1) $$\begin{array}{c} N_{i,j}=\frac{z_{i}}{z_{j}}, \end{array}$$

where *z* is the number of teeth and *i* and *j* are the leading and follower gears, and the equation relating joint movement *θ*_J1_ to the rotation *θ*_in,1_ can be expressed as follows:

(2) $$\begin{array}{c} \theta_{J1}=\theta_{\text{in},1}N_{reducer}N_{a1,a2}N_{a3,a4}, \end{array}$$

where *N*_reducer_ = 5.2, *N*_a1,a2_ = 1.88, and *N*_a3,a4_ = 20.

The input spur gear a1 is actuated by a brushless DC motor (MT8N42P06V2E), which is a 60 W motor coupled with the 5.2 : 1 gearhead, and the rated speed of the motor is 3000 RPM, while the rated torque is 0.2 Nm. By substituting the rated parameters into (2), the output of the robotic joint J1 can exert an angular velocity of 92 degrees/s. The output torque can be computed by

(3) $$\begin{array}{c} T_{1\text{out}}=T_{1}N_{reducer}N_{a1,a2}N_{a3,a4}\overset{4}{\underset{i=1}{\prod }}\eta_{i}, \end{array}$$

where *T*_1out_ is the output torque, *T*_1_ is the rated torque of motor I, *η*_1_ is the transmission efficiency of the planetary reducer and equals to 0.8, *η*_2_ is the transmission efficiency of the cylindrical gear pair and equals to 0.98, *η*_3_ is the transmission efficiency of the worm gear pair and equals to 0.75, and *η*_4_ is the transmission efficiency of the ball bearing and equals to 0.99. By substituting these parameters into (3), the output torque can exert a value of 22.76 Nm. Compared with the system specification, the mechanism design of the joint for internal/external rotation exercise can satisfy the requirement.

#### 2.3.2. Mechanism for Dorsiflexion/Plantarflexion Exercise

**Figure 5:**
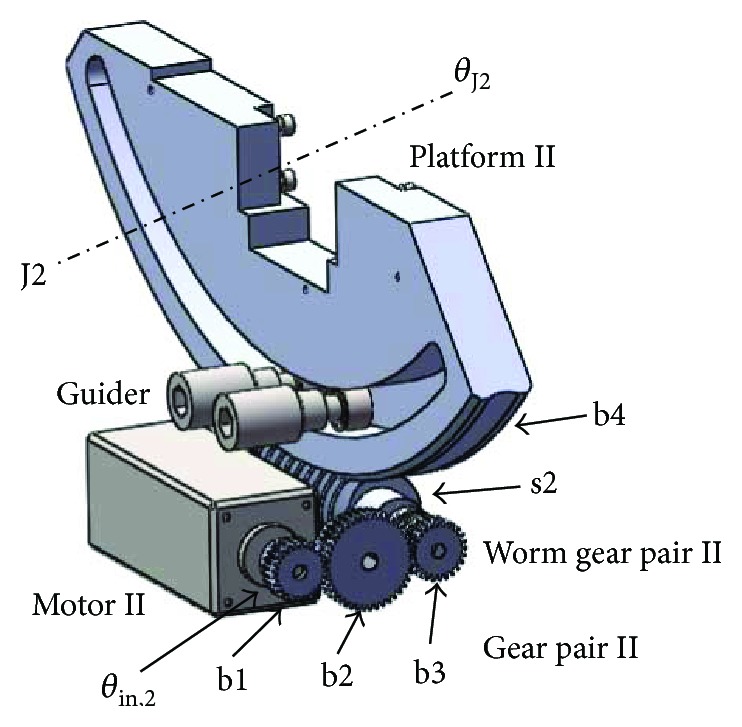
Components of the power transmission mechanism for dorsiflexion/plantarflexion exercise.

The mechanism for dorsiflexion/plantarflexion exercise, corresponding to J2 in Figure 5, is actuated by a brushless DC motor. The movement is transmitted from the motor II axis to the perpendicular joint axis by means of the combination of the spur gear pair and worm gear pair. The actuator is MT8N42P10V2E, able to provide 100 W of power with a rated torque of 0.32 Nm and a rated speed of 3000 RPM. The shaft of motor II is parallel with that of the worm rod, while executing power transmission though an intermediate wheel. Two guider rods are used to ensure that platform II works in the plate paralleled with shaft s2. The equation relating joint movement *θ*_J2_ to the rotation *θ*_in,2_ can be expressed as follows:

(4) $$\begin{array}{c} \theta_{J2}=\theta_{in,2}N_{b1,b2}N_{b2,b3}N_{b3,b4}, \end{array}$$

where *N*_b1,b2_ = 1.68, *N*_b2,b3_ = 0.595, and *N*_b3,b4_ = 172. By substituting the rated parameters of motor II into (4), the movement of the robotic joint J2 can execute a maximum angular velocity of 104.6 degrees/s. The output torque of the robotic joint J2 can be calculated by

(5) $$\begin{array}{c} T_{2\text{out}}=T_{2}N_{b1,b2}N_{b2,b3}N_{b3,b4}\left(\eta_{2}^{2}\eta_{3}\eta_{4}^{2}\right), \end{array}$$

where *T*_2out_ represents the output torque of motor II, *T*_2_ is the rated torque of motor II, *η*_2_ is the transmission efficiency of the cylindrical gear pair and equals to 0.98, *η*_3_ is the transmission efficiency of the worm gear pair and equals to 0.75, and *η*_4_ is the transmission efficiency of the ball bearing and equals to 0.99. By substituting these parameters into (5), platform II can execute a torque of 55.04 Nm, which can meet the design requirement.

#### 2.3.3. Mechanism for Inversion/Eversion Exercise

**Figure 6:**
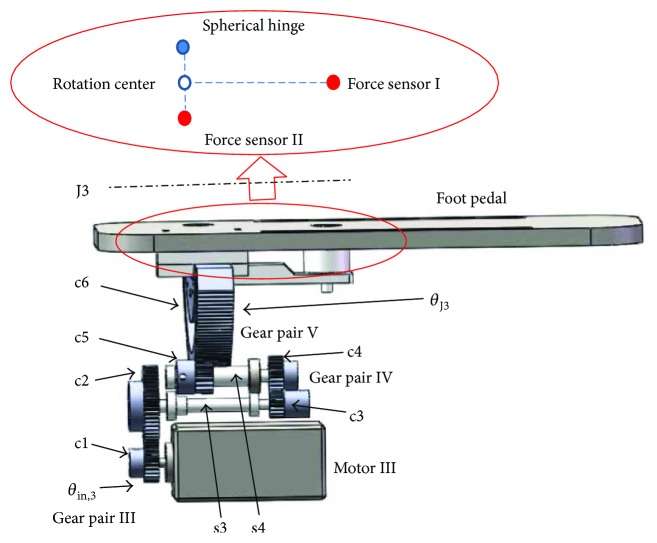
Components of the power transmission mechanism for inversion/eversion exercise.

As presented previously, the mechanism for inversion/eversion exercise is located at the top layer and fitted a foot pedal at the distal zone, as shown in Figure 6. Three gear pairs are employed to execute the power transmission from the motor shaft to the foot pedal. Gear c1 is mounted directly at the motor shaft. Gear c2 and gear c3 are mounted at the both sides of shaft s3 while gear c4 and gear c5 are installed at the both ends of shaft s4.

Gear c1, actuated by a brushless DC motor (MT8N42P06V2E), bites with gear c2 in gear pair III, and the similar configuration was constructed in gear pair IV and gear pair V. The equation bridging joint movement *θ*_J3_ to the rotation *θ*_in,3_ can be expressed as follows:

(6) $$\begin{array}{c} \theta_{J3}=\theta_{\text{in},3}N_{c1,c2}N_{c3,c4}N_{c5,c6}, \end{array}$$

where *N*_c1,c2_ = 2.2, *N*_b2,b3_ = 1.5, and *N*_b3,b4_ = 17.1. Based on the known parameters, we can obtain that the robotic joint J3 can achieve a maximum angular velocity of 319 degrees/s; the output torque of the robotic joint J3 can be described as

(7) $$\begin{array}{c} T_{3\text{out}}=T_{3}N_{c1,c2}N_{c2,c3}N_{c3,c4}\left(\eta_{2}^{3}\eta_{4}^{2}\right), \end{array}$$

where *T*_3out_ is the output torque of motor III, *T*_3_ is the rated torque of motor III, *η*_2_ is the transmission efficiency of the cylindrical gear pair and equals to 0.98, and *η*_4_ is the transmission efficiency of the ball bearing and equals to 0.99. By substituting the parameters into (7), the foot pedal can achieve 10.41 Nm for inversion/eversion exercise.

Two force sensors (FL25-100 kg, Forsentek Co., Shenzhen, China) are mounted under the foot pedal, based on the lever principle; the torque generated for dorsiflexion/plantarflexion and inversion/eversion movements can be calculated by

(8) $$\begin{array}{c} T_{inv\text{.}/ev.}=2F_{2}l_{1}+F_{1}l_{1},\\ T_{dor\text{.}/plantar.}=F_{1}l_{2}, \end{array}$$

where *T*_inv./ev._  and  *T*_dor./plantar._ represent the rotational torques of inversion/eversion and dorsiflexion/plantarflexion, respectively. *F*_1_ and *F*_2_ denote the measured force on sensor I and sensor II, and *l*_1_ and *l*_2_ are the length of the corresponding cantilevers.

### 2.4. Overview of the Control Architecture

**Figure 7:**
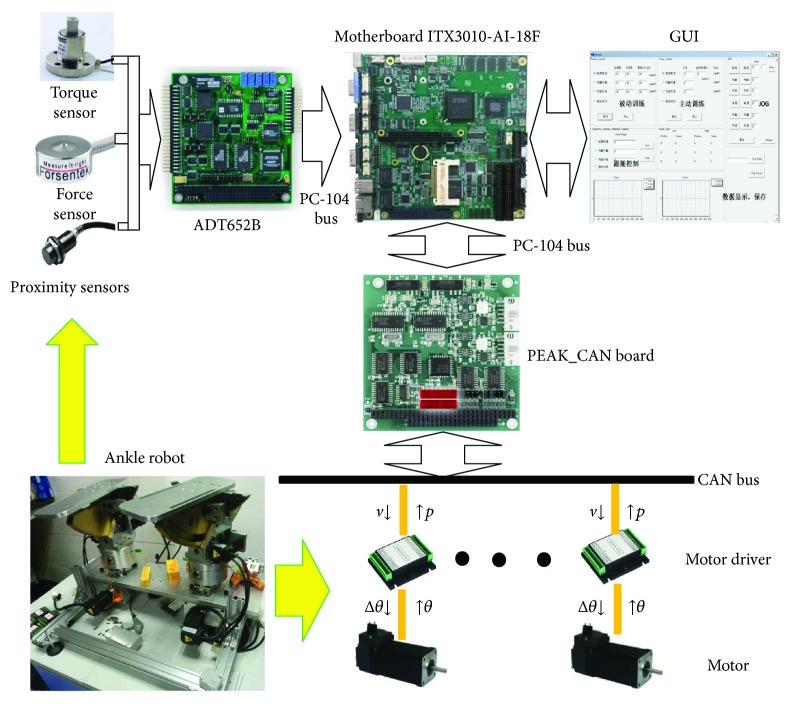
Control architecture of the ankle rehabilitation robotic system.

For the ankle rehabilitation robotic platform, six IBL3605A motor drivers (made by Techservo Co. Ltd., Shenzhen, China) are used to drive the two 3-DOF layer-stacking robotic platforms. Encodes embedded in the motors provide the current relative angular position of each motor shaft, thus enabling a semiclosed loop position control. All the drivers communicate via CAN bus with a PC-104-based personal computer (PC). The C++ software that runs on the PC obtains control instructions from the peripheral equipment (mouse, keyboard, and sensors) and sends the desired velocity of the motors to the controllers through a CAN-PC104 card (PEAK-System Technik, Germany). The relations between each submodule of the control system are described in Figure 7.

As shown in Figure 7, the user sets the ankle exercise parameters and chooses the exercise mode via GUI on the PC. By combining the sensing data and user input from GUI, the motion amount of each joint can be calculated in the PC and delivered to the corresponding motor through CAN bus. The generated encoder data, internal/external rotation torque (torque sensor), foot pressure (force sensor), and joint range limits (proximity sensor) will be sent back to the controller as a compensation for the input.

Based on the designed ankle rehabilitation robotic platform, a healthy subject with no experience of using the robot was selected to complete three trajectories on each exercise mode mentioned in Section 2.2. The user had 5 min to familiarize himself with the operating characteristics of the robot system before executing the ankle exercises.

## 3. Results and Discussion

As introduced in Section 2, the ankle rehabilitation robotic platform consists of two symmetric exercise mechanisms. The mechanical configuration allows patients to execute the affected ankle exercise only or the coordination training on both feet. Three ankle exercise modes are designed to execute ankle rehabilitation corresponding to different rehabilitation stages.

### 3.1. Constant-Speed Ankle Exercise

In the early stage of ankle therapy, the patient is unable to move his/her foot; hence, a patient-passive training which can delicately move the patient's foot is needed. In this stage, the patient's foot is unable to provide force to move the foot pedal. Therefore, the objective of this rehabilitation stage is to exercise the affected muscles to avoid muscle atrophy.

**Figure 8:**
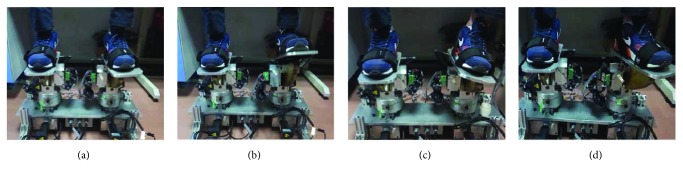
Experimental scenarios under patient-passive exercise mode: (a) initial posture; (b) dorsiflexion/plantarflexion exercise; (c) inversion/eversion exercise; (d) internal/external rotation exercise.

In order to avoid exercise injury, the robot should provide moderate exercise with a constant speed in the early stage. The experimental scenario is shown in Figure 8. The user sets the exercise velocity and angle of joint motion via GUI (parameters are listed in Table 3).

**Figure 9:**
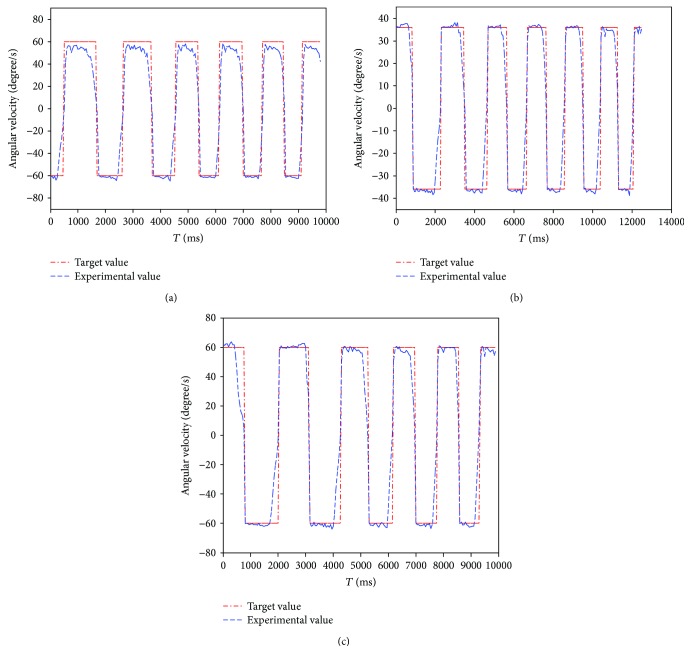
Experimental tracking result and setting parameters of patient-passive exercise: (a) dorsiflexion/plantarflexion movement; (b) inversion/eversion movement; (c) internal/external rotation.

The trail of joint velocity was recorded during exercise, and the relation between the experimental result and the target parameter is shown in Figure 9. The experimental result demonstrated that the robotic platform can well track the target value in the inversion/eversion movement and internal/external rotation. There is a mean error of 6°/s in the dorsiflexion movement, which is caused by the payload on the foot pedal due to motor impedance on the patient's ankle.

### 3.2. Constant Torque-Impedance Ankle Exercise

In this exercise mode, the patient lays his/her affected side foot on the foot pedal and executes force to push the foot pedal to enhance strength of the affected side ankle. A force threshold is set through GUI, and the foot pedal will move after the detected force on the foot pedal has exceeded the threshold value. The parameters under constant torque-impedance ankle exercise are noted in Table 4.

**Figure 10:**
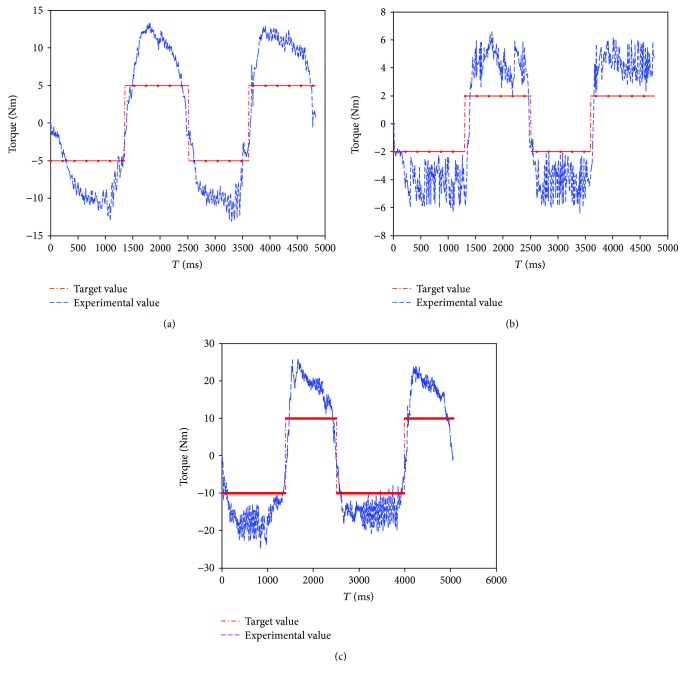
Experimental data and the target threshold under patient-active ankle exercise mode: (a) dorsiflexion/plantarflexion movement; (b) inversion/eversion movement; (c) internal/external rotation.

The experimental result under constant torque-impedance ankle exercise is shown in Figure 10. The robot joint will keep on moving when the detected force is smaller than the set threshold. The foot pedal moves with the velocity proportional to the deviation of the detected torque and the threshold value. The experimental result demonstrated that the foot pedal will quickly follow the movement of the patient's foot after the interaction torque has exceeded the threshold.

### 3.3. Awareness Exercise

**Figure 11:**
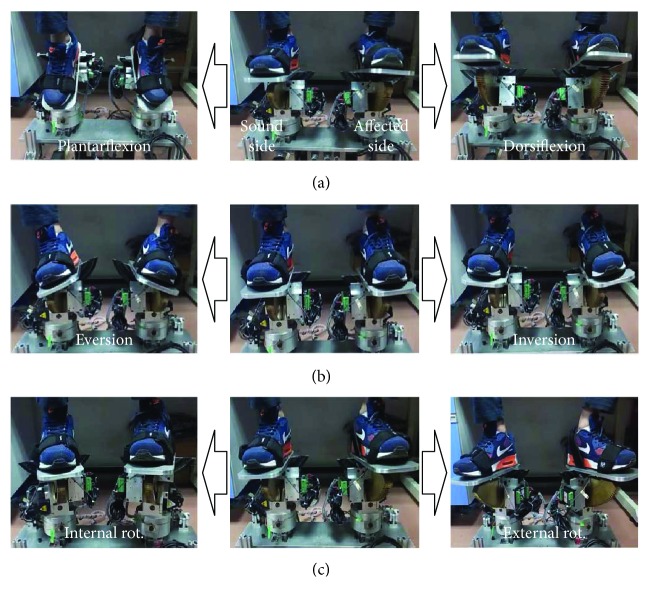
Ankle exercise under fusion of awareness and passive exercise modes: (a) movement of dorsiflexion/plantarflexion; (b) movement of inversion/eversion; (c) internal/external rotation.

In this training mode, the sound side of the patient's foot will lie on the foot pedal and drive the foot pedal to move in the ankle range of motion under self-awareness control. Since the mechanism of the affected side robotic platform is symmetric to that of the sound side, the movement of the sound side will be directly mapped to drive the mechanism at the affected side under position control. The experimental scenarios are shown in Figure 11.

**Figure 12:**
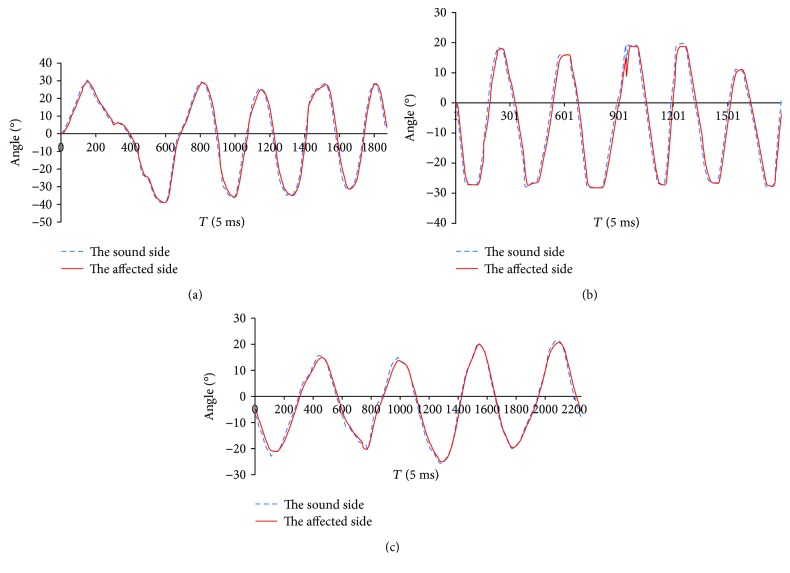
The ankle movement under awareness exercise mode: (a) dorsiflexion/plantarflexion; (b) inversion/eversion; (c) internal/external rotation.

Corresponding to the ankle exercise shown in Figure 11, the positioning tracking trajectories are described in Figure 12. The foot pedal at the sound side is driven by the patient under awareness exercise mode, and the foot pedal at the affected side follows well the trace generated at the sound side.

### 3.4. Discussion

The ankle exercises under constant-speed exercise mode are described in Figures 8 and 9. The experimental result demonstrated that the velocity of the ankle rehabilitation robotic platform follows well the input value. In the constant torque-impedance exercise mode, changing the threshold of the torque can realize strength training of the affected ankle. And the foot pedal of the robot will move with the velocity proportional to the deviation of the detected torque and the target torque. In the awareness exercise mode, the sound side of the robotic platform will teach the affected side to move in the range of motion. The root mean square (RMS) in the dorsiflexion/plantarflexion is 6.55 degrees, and the maximum time delay is 80 ms. The RMS in the inversion/eversion is 1.56 degree, and the maximum time delay is 70 ms. The RMS in the internal/external rotation is 1.58 degree, and the maximum time delay is 85 ms. The mean error and standard deviations are illustrated in Table 5.

The above-proposed three exercise modes correspond to different rehabilitation stages. In the constant-speed exercise mode, the patient cannot manage the affected muscle by himself/herself; therefore, the ankle rehabilitation robotic platform will move with a constant moderate speed with the consideration of individual difference, which aims to avoid patient muscle atrophy. The constant-speed exercise mode can be replaced by a torque resistance control scheme, which is used for enhancing the strength of the patient's ankle. In this mode, the ankle rehabilitation robotic platform is controlled to provide a certain level of torque to the footplate while the patient tries to fully regain his/her range of motion.

In the awareness rehabilitation procedure, the patient can exercise his/her affected side ankle using the motion data captured from the sound side ankle under proprioceptive training. In order to reobtain the individual balance, the patient has to lay his/her sound side ankle and affected ankle on the corresponding robotic platform, respectively, and then drive the sound side to move within the range of motion under his/her awareness, while the affected side ankle robotic platform will track the same trail mapped from that of the sound side ankle robot to exercise the affected ankle.

The experimental results demonstrated that the ankle rehabilitation robotic platform is capable of executing the above three exercise modes. Based on the experimental results, several improvements are needed to be addressed as follows.

#### 3.4.1. A Friendly Interface for the Robot User

Currently, robot users input instructions to the control system via a mouse and keyboard. A touch panel, integrated into the control architecture, will enable the robot user to easily enter the parameters and instructions. Furthermore, the touch panel is portable, which allows the user to set the rehabilitation parameters at a comfortable location.

#### 3.4.2. Integration with Virtual Reality Scenarios

Although patients can perform ankle training under awareness exercise mode, a virtual training scenario integrated with patient rehabilitation information will stimulate the robot user to concentrate on training and make the rehabilitation become an attractive activity.

## 4. Conclusions

This paper presented a novel layer-stacking structure robotic platform used for robot-aided ankle rehabilitations. The ankle rehabilitation robot consists of two symmetric 3-DOF robotic platforms, where one is used to detect the movement of the sound side ankle and the other is used to exercise the affected side ankle. The unique mechanical configuration allows the patient to exercise his/her affected side ankle via the movement taught by the sound side ankle. The rehabilitation protocol has been considered the basis for design of the control architecture. Based on the designed robotic system, three exercise modes including constant-speed exercise, constant torque-impedance exercise, and awareness exercise modes have been developed to perform ankle training corresponding to different rehabilitation stages. The experimental results demonstrated that the promising performance of tracking trail between the two symmetric robotic platforms was obtained with a mean tracking error of 6°/s under constant-speed exercise mode. The robotic platform can move smoothly in the constant torque-impedance mode, and the robotic platform at the affected side can well track the movement of the sound side with the maximum mean error of 6.55°.

Future work will look at the development of a friendly human-machine interface and the integration of a virtual environment based on the robot's vision to stimulate the patient during training [16]. Rehabilitation experiments with a team of clinicians are under preparation. Further studies will also extend to the integration of the EMG and FES information in the control architecture.

## Figures and Tables

**Table 1:** Existing state-of-the-art ankle rehabilitation robots.

Catalog	System or developer	DOFs	Payload
Parallel structure	Saglia et al. [2]	2	≤120 Nm
	Rutgers University [5]	6	≤35 Nm
	Liu et al. [6]	3	
	Meng et al. [7]	6	—
	Muhammad and Shafriza [8]	3	—
	Yu et al. [9]	3	—

Exoskeletal structure	Jeffrey et al. [10]	1	≤30 Nm
	Delaware University [11]	2	—
	Rahman and Ikeura [12]	1	≤60 Nm
	Hong et al. [13]	3	—

**Table 2:** Ankle physiological data.

Axis	Motion	Angle range (degree)	Torque (Nm)	Angular velocity (degrees/s)
*x*	Inversion	0~30	10	≤100
	Eversion	−20~0		

*y*	Dorsiflexion	0~30	45	≤80
	Plantarflexion	−40~0		

*z*	Internal rot.	0~20	20	≤80
	External rot.	−30~0		

**Table 3:** Parameters for constant-speed ankle exercise.

Parameter joint	Angular velocity (degrees/s)	Range (degrees)
Dor./plantar.	60	−30~30
Inv./ev.	36	−20~20
Int./ex. rot.	60	−20~20

**Table 4:** The setting parameters under constant torque-impedance ankle exercise.

Movement	Dorsiflexion/plantarflexion	Inversion/eversion	Internal/external rotation
Torque (Nm)	5	2	10

**Table 5:** The mean error and standard deviations on the three exercise modes.

Exercise mode 1	Trail			Mean error	Standard deviation
	1	2	3		

Dorsiflexion/plantarflexion (°/s)	6.6	6.3	5.1	6	0.79
Inversion/eversion (°/s)	1.2	0.6	0.9	0.9	0.3
Internal/external rotation (°/s)	1.2	0.6	0.9	0.9	0.3

Exercise mode 2	Trail			Mean error	Standard deviation
	1	2	3		

Dorsiflexion/plantarflexion (Nm)	6.2	5.8	6.4	6.13	0.31
Inversion/eversion (Nm)	3.6	3.2	3.5	3.43	0.21
Internal/external rotation (Nm)	8.6	8.1	8.3	8.33	0.25

Exercise mode 3	Trail			Mean error	Standard deviation
	1	2	3		

Dorsiflexion/plantarflexion (°)	6.94	6.82	5.89	6.55	0.57
Inversion/eversion (°)	1.69	1.47	1.52	1.56	0.12
Internal/external rotation (°)	1.65	1.47	1.62	1.58	0.10
